# An Intelligent Tongue Diagnosis System via Deep Learning on the Android Platform

**DOI:** 10.3390/diagnostics12102451

**Published:** 2022-10-10

**Authors:** Zibin Yang, Yuping Zhao, Jiarui Yu, Xiaobo Mao, Huaxing Xu, Luqi Huang

**Affiliations:** 1 School of Electrical and Information Engineering, Zhengzhou University, Zhengzhou 450001, China; 2 China Academy of Chinese Medical Sciences, Beijing 100020, China

**Keywords:** mobile terminal, tongue, intelligence, inference, deep learning

## Abstract

To quickly and accurately identify the pathological features of the tongue, we developed an intelligent tongue diagnosis system that uses deep learning on a mobile terminal. We also propose an efficient and accurate tongue image processing algorithm framework to infer the category of the tongue. First, a software system integrating registration, login, account management, tongue image recognition, and doctor–patient dialogue was developed based on the Android platform. Then, the deep learning models, based on the official benchmark models, were trained by using the tongue image datasets. The tongue diagnosis algorithm framework includes the YOLOv5s6, U-Net, and MobileNetV3 networks, which are employed for tongue recognition, tongue region segmentation, and tongue feature classification (tooth marks, spots, and fissures), respectively. The experimental results demonstrate that the performance of the tongue diagnosis model was satisfying, and the accuracy of the final classification of tooth marks, spots, and fissures was 93.33%, 89.60%, and 97.67%, respectively. The construction of this system has a certain reference value for the objectification and intelligence of tongue diagnosis.

## 1. Introduction

Tongue diagnosis is an important part of inspection in Traditional Chinese Medicine (TCM) that was recognized by the World Health Organization (WHO) in 2018 [1]. The appearance of the tongue conveys an array of valuable information for medical diagnosis in Western and Oriental medicine. Abnormalities in tongue color and texture are commonly examined by medical professionals for either health status checks or disease diagnosis. In Western medicine, a tongue fissure is a typical texture malformation found to be closely associated with Melkersson Rosenthal syndrome [2], Down’s syndrome [3], diabetes [4], and some other kinds of diseases. In Oriental medicine, TCM practitioners can discern the deficiency and excess of viscera, pathological states, and the region of disease by observing tongue features such as the color, fur, tooth marks, fissures, degree of moisture, and spots. Visual inspection of the tongue can offer an immediate, simple, cheap, and convenient solution for medical analysis [5].

However, this is limited by the fact that the clinical competence of a tongue diagnosis depends heavily on the experience and ability of the TCM practitioner. The diagnostic results based on the subjective analysis of the examiners may be unreliable and inconsistent. Therefore, it is important to have an objective and quantitative diagnostic process for tongue diagnosis. To address this issue, the integration of computer science and tongue diagnosis is becoming a key research direction in the field of intelligent tongue diagnosis.

Recently, with the rapid development of image processing, tongue diagnosis has made great progress in terms of tongue image processing and feature analysis. Among the deep learning image processing techniques used [6,7,8], the convolutional neural network can learn how to detect the tongue body from pictures, segment the tongue region, which may reduce the influence of elements in the external environment such as the teeth and cheeks in subsequent steps, and can also learn how to extract the characteristics of the tongue so as to assist doctors with tongue diagnosis.

In the past few years, some tongue classification studies and computer-aided tongue diagnosis systems [9,10,11] have employed deep learning technology to quantify the color channels and texture features of the tongue body or fur to diagnose different diseases. The authors of ref. [9] presented an automatic disease detection system based on a multi-view instance (face, tongue, and sublingual vein) captured from an individual. The authors of [10] used computer tongue image analysis technology to construct different nonalcoholic fatty liver disease (NAFLD) diagnostic models to find the best diagnostic model suitable for large-scale NAFLD screening. The authors of [11] proposed a method using the surface and color features of tongue based on convolutional deep neural networks to increase the diagnosis precision of gastric cancer, as well as a support vector machine. These systems have mainly been developed based on computers or other embedded devices, so their real-time performance and portability are insufficient, which limits the application of intelligent tongue diagnosis systems to some extent.

In addition, some tongue processing algorithm studies have only focused on detection [12], segmentation [13,14], or classification [15,16]. The authors of [12] used a one-stage detector SSD with MobileNetV2 to detect tongue regions. The authors of [13,14] proposed a new end-to-end tongue localization and segmentation method and a fast tongue segmentation system based on U-Net. The authors of [15] explored the convolutional neural network method in order to classify tongue color from tongue images, and in [16], a multiple-instance method was presented for the recognition of tooth-marked tongues. Though these studies have made some progress, they are all independent, and there is no tongue system on the Android platform that integrates all three modules simultaneously.

Based on the aforementioned observations, we propose and develop a tongue diagnosis system that includes registration, login, account management, tongue recognition, and doctor–patient dialogue modules on the Android platform. The whole system uses a smartphone as the platform for collecting tongue images and presenting the results. The detection model is deployed on a smartphone to recognize the tongue when the user prepares to take a picture of the tongue, and other models (segmentation and classification) are used for tongue image processing. Finally, the system presents a tongue diagnosis report regarding the tongue diagnosis results and treatment recommendations for users. With the collected tongue image datasets, the conducted experiments demonstrate that our tongue diagnosis system can achieved a convenient, intelligent, and objective tongue diagnosis, and the idea presented here can act as a reference for the development of intelligent and objective tongue diagnosis methods.

The remainder of this paper is organized as follows. In Section 2, we describe the system, including its architecture, some basic models, the diagnosis process, physical information collection, and “asking doctors” in detail. In Section 3, we discuss the methods selected for detection, segmentation, and classification. In Section 4, the experiments are presented, which include data splitting, the training set-up, and model evaluation. In Section 5, the discussion and future work are presented.

## 2. System

### 2.1. Architecture

**Figure 1:**
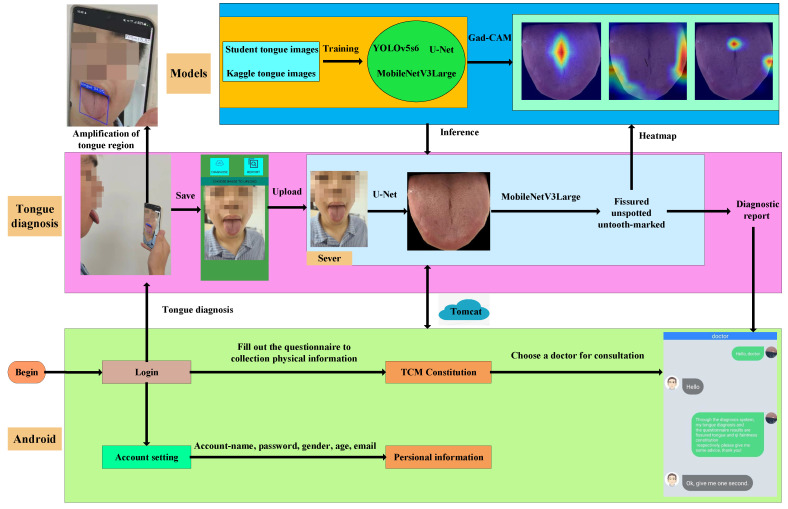
The architecture of the proposed tongue diagnosis system.

Our system is comprised of two main parts: the mobile terminal and the cloud server. The user is instructed to take a photo with a mobile phone or select an existing tongue image from the photo gallery and upload it to the cloud server. Then, the related models and algorithms process the images and analyze the tongue features to generate treatment recommendations. Finally, the results and recommendations are fed back in the form of a diagnosis report. Figure 1 shows the architecture of the proposed tongue diagnosis system.

The development environment used for this system is JDK1.8+Android studio+lntelliJ IDEA, where the Android studio is the mobile system development platform and IntelliJ IDEA is used to run the logic on the cloud server. The system uses Tomcat [17] as the software server to act as the data transfer pipeline between the mobile terminal and the cloud server.

### 2.2. Basic Modules

As a typical Android system, our system includes some basic modules: registration, login, and account settings. The basic module usage is as follows. First, the user enters his or her details, including an account name, password, gender, age, and email, to register an account. Then, the information can be used to log in. Inside the system, there are three fragments (the interfaces of the mobile app), including tongue diagnosis, a questionnaire and option to ask a doctor, and account settings, where the user can modify the personal information registered.

### 2.3. Diagnosis Process

**Figure 2:**
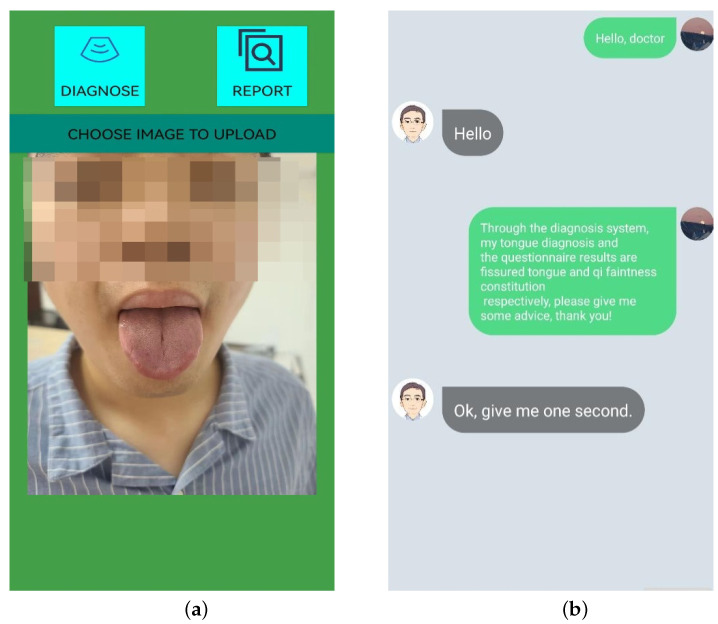
Interfaces of the tongue system. (**a**) The interface to upload tongue images. (**b**) The dialog window.

This part is the core of the system. It includes taking pictures, uploading and processing tongue images, and generating the tongue diagnosis report. The user is instructed to ensure he or she is under natural light or a standard D65 light source created to simulate natural light to take pictures of the tongue. Then, the user can choose a tongue image from the album to upload, click on the diagnosis button, and receive a tongue diagnosis report. Figure 2a,b shows the tongue diagnosis interface and the tongue diagnosis report.

### 2.4. More Physical Information and “Asking Doctors”

**Figure 3:**
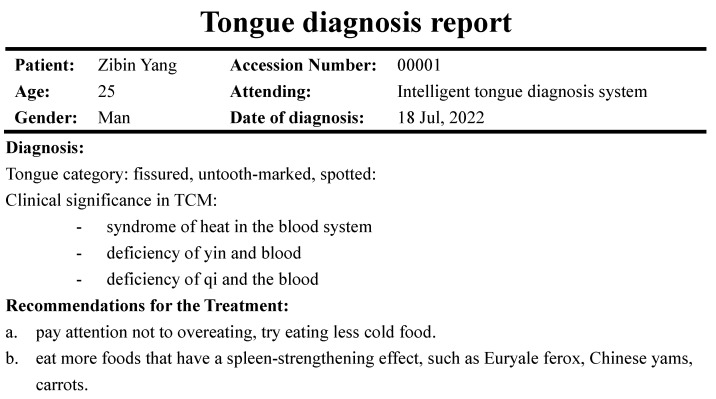
Tongue diagnosis report.

The system includes an online TCM constitution assessment system with a questionnaire form that was developed according to the standards of the Chinese Society of Traditional Chinese Medicine. It is used to collect more physical information about the user to supplement the tongue diagnosis. After the questionnaire, the user can select a doctor to receive a more detailed consultation based on the results of the questionnaire and the tongue diagnosis report. Figure 3 shows a window with doctor–patient dialogue.

## 3. Tongue Processing Framework

Our framework includes three lightweight network models: YOLOv5s6, U-Net [18], and MobileNetV3Large [19]. YOLOv5s6 detects whether a picture contains the tongue. U-Net segments the tongue region to eliminate the effect of the face and other background areas. MobileNetV3Large is used to classify the tongue’s features.

### 3.1. Tongue Detection

There are three steps used in traditional image detection methods: region selection, feature extraction, and feature classification. These methods have poor precision and generalization.

Modern object detection algorithms use a deep learning model to extract features, which preserves image information well, and the accuracy and robustness are greatly improved compared with traditional algorithms. They can generally be assigned as single-stage detection or multi-stage detection methods. Single-stage methods are fast and have good real-time performance, which is not needed to identify candidate regions. However, the level of accuracy is low. Such methods include YOLO [20,21,22,23] and SSD [24,25,26,27]. Multi-stage models can achieve high-accuracy levels, but they are slow. Multi-stage methods work similarly to traditional algorithms. First, the candidate regions are obtained, and then the classifiers are used for classification. Such models include R-CNN [28], Fast R-CNN [29], Faster R-CNN [30], and Mask R-CNN [31]. Compared with SSD, YOLO has obvious advantages in terms of its recognition speed and accuracy. This system uses the latest sixth version of its fifth version of YOLO, YOLOv5s6, as the detection network, which meets the requirements of the system in terms of accuracy and real-time performance.

**Figure 4:**
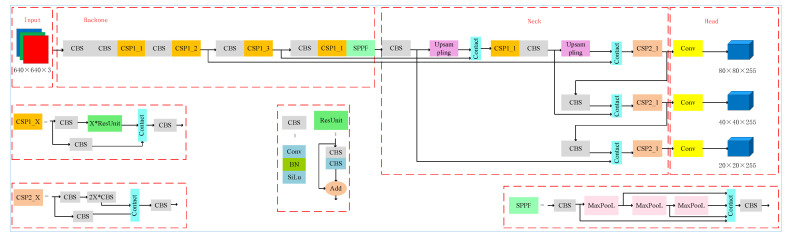
The architecture of YOLOv5s6.

Figure 4 shows the architecture of YOLOv5s6 and some special modules. The network is composed of four main parts: the input, backbone, neck, and head. The input module is used for resizing raw images. The backbone network includes CSP1_X (where X is the number of the ResUnit), CBS (conv + BN + SiLU, as seen in Equation (1)), and the SPPF layer, a variant of spatial pyramid pooling (SPP) [32]. The feature pyramid network [33] (FPN) and path aggregation network [34] (PAN) are used in the neck model. Meanwhile, the neck contains CSP2_X (where 2×X is the number of CBS) and some standard convolution layers. The last model is the head, which is designed to carry out predictions. Three pipelines are used to detect objects at different scales:

(1) $$SiLU\left(x\right)=x\times \frac{1}{1+e^{-x}}$$

First, the tongue image is resized to 640×640 pixels. Then, the backbone extracts the tongue region features, and the neck is used for the sampling and fusion of feature maps by the FPN [33] and PAN [34]. Finally, the detection result is presented by the head. The result includes a 3D tensor encoding a bounding box, objectness, and category predictions [22].

### 3.2. Tongue Region Segmentation

The traditional image segmentation algorithm is mainly based on the pixel value of the image. The pixel values of the image in particular regions have a certain degree of similarity and strong correlations, while the pixel values at the edges of different regions are discontinuous. However, the principle of these algorithms is that they must be simple, robust, and accurate, making it difficult to meet the practical application requirements.

Deep learning algorithms can extract middle- and high-semantic information from images and obtain precise semantic segmentation results. The classic semantic segmentation algorithms include the FCN [35], U-Net [18], and DeepLab [36,37,38,39]. U-Net is widely used in the field of medical image segmentation. Compared with other networks, it is more accurate, has fewer network parameters, and has better real-time performance, allowing it to meet the needs of mobile segmentation tasks.

**Figure 5:**
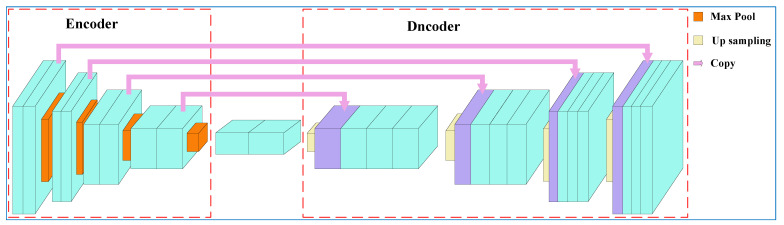
The architecture of U-Net.

U-Net is a fully convolutional network consisting of two parts, as shown in Figure 5: the encoder and the decoder. The image is first resized to 480×480 pixels, and then the encoder extracts and compresses the features from the image using multiple convolution layers and max pooling layers (downsampling). Finally, it obtains feature maps 30×30×1024 pixels in size. Then, the decoder combines the bilinear (upsampling) and convolution layers to predict a binary image (the pixel value of the tongue region is 1, and the value of the other pixels is 0) 480×480 in size. There are some pipelines to transmit features and superimpose them on subsequent layers to enhance the information and resolution of the neural networks between the encoder and decoder [40].

### 3.3. Tongue Feature Classification

Tongue feature classification can be regarded as a typical image classification task. There are many classification networks that perform well in the field of deep learning, such as the VGG [41], ResNet [42,43,44,45], and MobileNet [19,46,47].

MobileNets are a family of mobile-first computer vision models developed by Google. MobileNets are all based on depthwise separable convolution, which factorizes a standard convolution into a depthwise convolution and a 1 × 1 convolution called a pointwise convolution. The depthwise convolution uses a filter for each channel of the former layer’s input, and the pointwise convolution applies a 1 × 1 convolution to combine the outputs of the feature maps.

**Figure 6:**
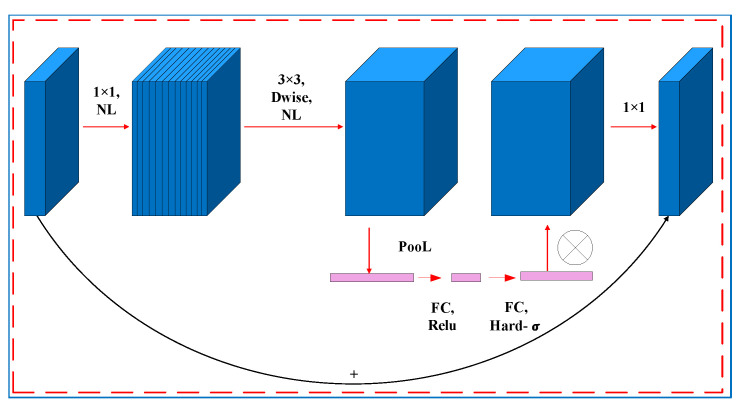
The bneck structure of MobileNetV3.

There are three versions of the MobileNet model: MobileNetV1, V2, and V3. MobileNetV1 [46] uses width and resolution multipliers to provide a balance between accuracy, computational latency, and model size. MobileNetV2 [47] applies linear bottlenecks with inverted residuals and is designed to have better memory-efficient inference. MobileNetV3 [19], the model used in our study, provides improved performance compared with the other models and includes the Squeeze and Excitation (SE) attention module in the bottleneck (bneck), the activation function (H–swish instead of Relu), and a redesigned expensive layer. These features allow it to achieve a faster inference speed and higher accuracy than the previous versions. Figure 6 shows the bneck structure of MobileNetV3. Table 1 shows the precise layout of MobileNetV3Large.

The process of inference is as follows. First, the model resizes the tongue image to 224×224 pixels and then extracts the feature with a standard convolution and 15 bnecks. Finally, the average pooling layer and three standard convolution layers are used for further inference to obtain the label of the image.

### 3.4. Evaluation Metrics

In deep learning, a confusion matrix [48] is a 2×2 (the number of target classes) matrix used for evaluating the performance of a machine learning model, where the rows represent the prediction outcomes and the columns represent the actual values. The meanings of the four basic terminologies (TP, FP, FN, and TN) are as follows:

- TP: true positive, where the actual value is positive and the predicted value is also positive;
- FP: False positive, where the actual value is negative and prediction is also negative;
- FN: false negative, where the actual value is negative but the prediction is positive;
- TN: true negative, where the actual value is positive but the prediction is negative.

**Tongue Detection**

The metrics used to evaluate tongue detection are the precision (Equation (2)), recall (Equation (3)), mean average precision (mAP) (Equation (6)), and the variants of the mAP. The mAP is the mean value for the average precision of each class, which is defined as the area under the precision–recall (Equation (5)) curve obtained by the sampling precision and recall, while k presents the number of tongue feature categories:

- Precision: This metric indicates the performance with respect to the false positives (i.e., how many the model identified);
- Recall: This metric indicates a classifier’s performance with respect to the false negatives (i.e., how many the model missed);
- IoU: The IoU (Equation (4)) is a standard for defining the detection accuracy of the target objects. The IoU evaluates the performance of the model by calculating the overlap ratio between the predicted bounding box and the true bounding box. $S_{overlap}$ is the area of intersection of the predicted bounding box and the true bounding box. $S_{union}$ is the area of the union of the two bounding boxes. The IoU threshold is a judgment criterion. If the IoU of the object is bigger than the threshold, then the object is thought of as a TP; otherwise, it is an FP.
- mAP@0.5: The parameter of 0.5 means that the threshold of the IoU is set to 0.5. Accordingly, the corresponding APs of all pictures of each category are computed and averaged.
- mAP@0.5:0.95: The parameters of 0.5:0.95 mean that the threshold of the IoU is increased from 0.5 to 0.95 with an increment of 0.05, and then each mAP is calculated by Equation (6). Finally, mAP@0.5:0.95 is the average of all mAPs:

(2) $$Precision=\frac{TP}{TP+FP}$$

(3) $$Recall=\frac{TP}{TP+FN}$$

(4) $$IoU=\frac{S_{overlap}}{S_{union}}$$

(5) $$AP=\int_{0}^{1}P\left(R\right)dR$$

(6) $$mAP=\frac{\sum_{i=1}^{k}AP^{i}}{k}$$

**Tongue Region Segmentation**

The metrics commonly used to evaluate tongue segmentation models are the mean pixel accuracy (MPA) (Equation (7)) and mean intersection over union (MIoU) (Equation (8)). The formulas used for tongue segmentation can be defined as follows, where k is the number of pixel categories:

- MPA: the average classification accuracy for each pixel category;
- MIoU: the mean value of the intersection over union, which is a very straightforward metric that is extremely effective for semantic segmentation:

(7) $$MPA=\frac{1}{k+1}\sum_{i=0}^{k}\frac{TP+TN}{TP+FN+FP+TN}$$

(8) $$MIoU=\frac{1}{k+1}\sum_{i=0}^{k}\frac{TP}{FN+FP+TP}$$

**Tongue Feature Classification**

Accuracy (Equation (9)), specificity (Equation (10)), F1-score (Equation (11)), precision (Equation (2)), and recall (Equation (3)) are often used as evaluation metrics for the classification of tongue features, where k is the number of tongue feature categories:

- Accuracy: a good measure when the target variable classes in the data are nearly balanced;
- Specificity: a measure that tells us the proportion of negative values that were predicted by the model as TN, which is the exact opposite of the recall;
- F1-Score: an efficient measure that combines precision and recall into a single metric and can give a larger weight to categories of lower numbers so it can be more objective for unbalanced datasets:

(9) $$Accuracy=\frac{1}{k+1}\sum_{i=0}^{k}\frac{TP+TN}{TP+FN+FP+TN}$$

(10) $$Specificity=\frac{TN}{TN+FP}$$

(11) $$F1-score=\frac{2\times Precision\times Recall}{Precision+Recall}$$

## 4. Model Training

### 4.1. Data Acquisition

**Figure 7:**
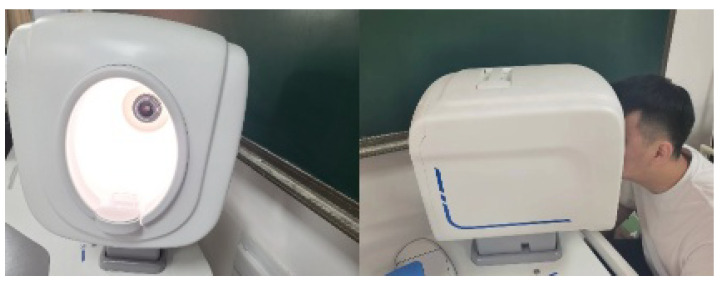
The device used for acquiring tongue images.

**Figure 8:**
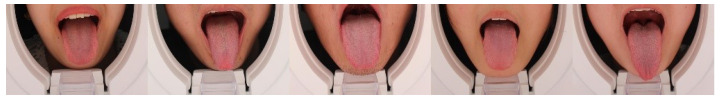
Some samples of dataset 1.

**Figure 9:**
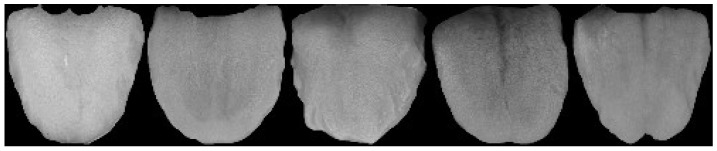
Some samples of dataset 2.

To train efficient and robust tongue diagnosis models, two datasets were used for training and testing in the experiments. The first dataset was acquired by volunteers, mainly consisting of college students, using a Canon Eos 700d camera in an enclosed environment (standard D65 light source built inside the device). During collection, the volunteers were instructed to naturally stretch out their tongues and ensure that their tongues were about 30–40 cm from the camera. Figure 7 shows the tongue image capture device. A total of 462 RGB 3-channel images were collected with a pixel size of 1728×2592. Then, the dataset was labeled by five expert TCM practitioners from the China Academy of Chinese Medical Sciences. If more than half of the experts thought a label was right, then the label was treated as the actual ground truth. Ten subdatasets were created based on their characteristics, including five tongue fur and body features, as shown in Table 2. However, due to the serious lack of clinical samples for some tongue features, they could not be fully used to train the tongue diagnosis model. Finally, only the samples with relatively balanced fissures and spots were selected for the training datasets. Dataset 2 is available on the Kaggle website. It contains 564 tooth-marked tongues and 704 unmarked tongue images. Figure 8 and Figure 9 shows some samples of datasets 1 and 2.

### 4.2. Data Preparation

The tongue image was initially cropped to 1728×1100 pixels in size (the bottom was retained) before the experiments, because the tongue image captured by the device was too large and contained a lot of useless information. In addition, the labels for detection and segmentation were elaborated by TCM practitioners with the aid of labelme [49] and labelimg [50]. The training set and testing set were produced by randomly splitting each dataset into proportions of 80% and 20%, respectively. Meanwhile, some data augmentation tricks were used to expand the training set and alleviate the overfitting of the model: (1) random vertical or horizontal flipping and (2) random rotation by $90^{\circ }$, $180^{\circ }$, and $270^{\circ }$. Table 3 shows the number of each task image used for training and testing.

### 4.3. Experiments and Training Set-up

Our experiments were performed on a server (Intel(R) Xeon(R) Gold 5218 CPU, 128 GB RAM, NVIDIA GTX 2080Ti graphic card) running the operating system Ubuntu Linux 21.04. All models were created in the Python programming language (python3.7.10) using Pytorch 1.11.0 and CUDA 11.4 for model compilation and training.

During training, a transfer learning trick based on the official Pytorch pretraining model was used as a benchmark. The networks using mini-batch SGD were trained with a learning rate set to 0.05, a momentum of 0.9, and a weight decay of 0.0001. The other precise parameters used can be found in Table 4. In addition, we used Cosine (Equation (12)) as the policy for the learning rate in all tasks and cross-entropy as the loss function for segmentation and classification. Its equation is as follows:

(12) $$Cosine=min\_lr+\left(initial\_lr-min\_lr\right)*(\left(1+cos\left(\frac{curr\_epoch}{epoch}*pi\right)\right)/2)$$

Cosine represents the newly obtained learning rate, initial_lr and min_lr are the ranges for the learning rate, where min_lr represents the minimum learning rate and initial_lr represents the initial learning rate, curr_epoch represents the current training epoch, and epoch is the the total number of training epochs [51].

The loss function of YOLOv5s6 is in [52] and consists of three parts: the confidence loss $l_{object}$, the classification loss $l_{class}$, and the position loss of the target box and the prediction box $l_{box}$. The calculation equation is as follows:

(13) $$loss=l_{obj}+l_{class}+l_{box}$$

The confidence loss $l_{obj}$ is used only to calculate the positive sample loss, but the classification loss $l_{class}$ calculates the loss of all samples. They all deploy binary cross-entropy loss (BCELoss) [53] as a loss function.

The CIoU [54] is used as the regression loss function of the tongue detection task. It can be expressed as

(14) $$l_{box}=l_{CIoU}=1-IoU+\frac{\rho^{2}\left(b,b^{gt}\right)}{c^{2}}+\alpha \upsilon$$

where *b* and $b^{gt}$ represent the central points of the predicted box and target box, ρ is the Euclidean distance between *b* and $b^{g}t$, and *c* is the diagonal length of the smallest enclosing box covering the boxes. In Equations (15) and (16), υ judges the consistency of the aspect ratio, and α is a positive tradeoff parameter. The formulae for these are as follows:

(15) $$\upsilon =\frac{4}{\pi^{2}}{\left(arctan\frac{\omega^{gt}}{h^{gt}}-arctan\frac{\omega }{h}\right)}^{2}$$

(16) $$\alpha =\frac{\upsilon }{(1-IoU)+\upsilon }$$

### 4.4. Results

**Figure 10:**
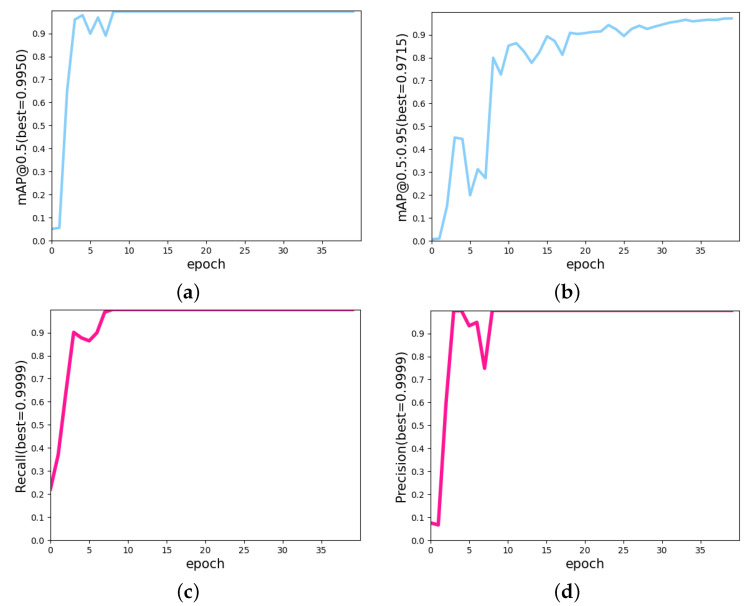
The metrics of the yolov5 change curve. (**a**) The curve of the change in mAP@0.5. (**b**) The curve of the change in mAP@0.5:0.95. (**c**) The recall change curve. (**d**) The precision change curve.

We can see that mAP@0.5 and mAP@0.5:0.95 of YOLOv5s6 could achieve values of 99.50% and 97.15%, respectively, after several epochs, and the precision and recall values reached 99.99%, as shown in Figure 10. This demonstrates the superior performance of YOLOv5s6 for the tongue dataset.

**Figure 11:**
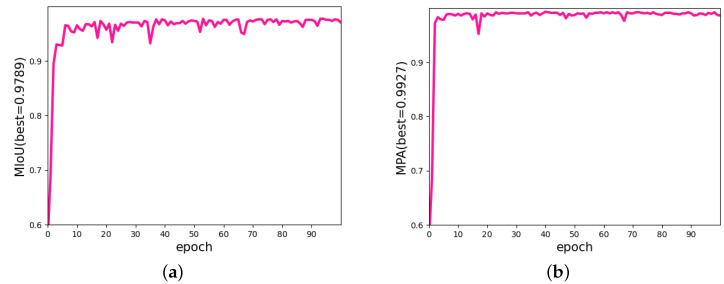
The metrics of the U-Net change curve. (**a**) The curve of the change in MIoU. (**b**) The curve of the change in MPA.

Figure 11 shows the U-Net training curve, where the MIoU and MPA values are up to 97.86% and 99.10%, respectively. This result means there was almost no difference between the true and predicted tongue areas, and the effectiveness can also be proven in the subsequent chapters.

**Figure 12:**
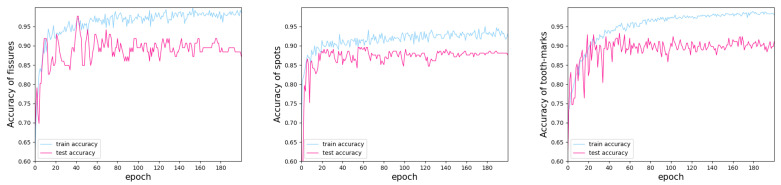
The accuracy change curve for MobileNetV3Large for different features.

The accuracy curves of different tongue datasets are shown in Figure 12. The accuracy change curves for the fissured and spotted datasets were unstable in the early epoch, while on the contrary, the change curve of the tooth-marked datasets only had small fluctuations. This could be because there were less data for the fissured and spotted samples than for the samples with tooth marks. Overall, although the training curves of the three datasets were somewhat different, they all gradually converged and achieved satisfactory results. Table 5 expresses the highest values for accuracy and other indicators for the same epoch. Compared with the samples with tooth marks and spots, the accuracy of the fissured samples was better (97.67%), which indicates that MobileNetV3Large is useful for extracting fissured features. Although the results for the tooth marks and spots were worse than those of the fissured samples, they still basically met our demands (tooth-marked: 93.33%; spotted: 89.60%).

### 4.5. Model Evaluation

To verify the feasibility of the models (YOLOv5s6, U-Net, and MobileNetV3Large), the three models were tested using some tongue images from the test datasets in different ways.

**Figure 13:**
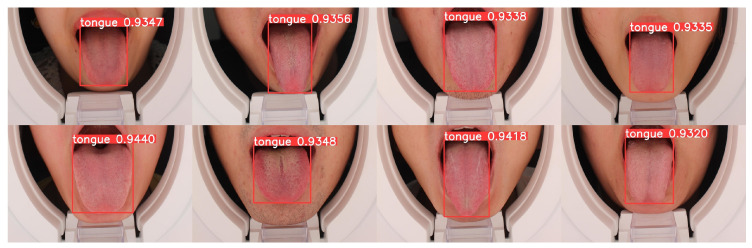
The detection results for the four tongue images from YOLOv5s6.

First, 10 images were used to estimate the detection model, and the results are shown in Figure 13. The bounding boxes predicted by YOLOV5s6 surrounded the tongue body perfectly with a high probability (>93%), thereby proving the effectiveness of YOLOv5s6.

**Figure 14:**
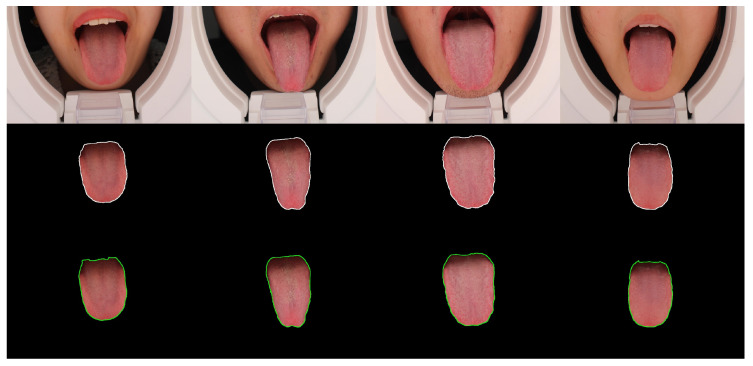
The segmented results for the four tongue images produced by U-Net. The white and green lines indicate the segments labeled by TCM practitioners and the trained model.

Secondly, Figure 14 shows three rows of images. The first, second, and third rows represent the raw, manually annotated, and model-annotated images, respectively. We used different colored lines to distinguish between them because the difference between the predicted and true values cannot be seen with the human eye, which verifies the admirable performance of the segmentation model.

**Figure 15:**
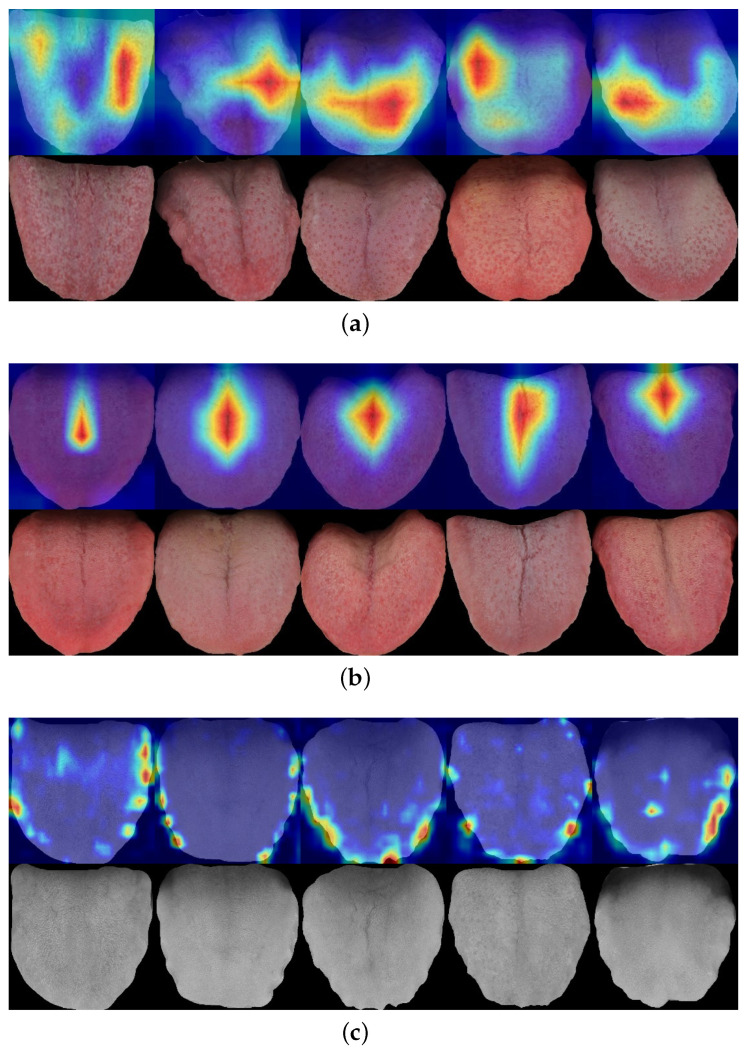
Heat maps of different features: (**a**) spotted tongue, (**b**) fissured tongue, and (**c**) tooth-marked tongue.

Third, we used gradient-weighted class activation mapping (Grad-CAM) [55] to create heat maps of the models. Grad-CAM is a popular technique for visualizing convolutional neural network models. Figure 15a–c shows the heat maps of the spotted, fissured, and tooth-marked tongues, respectively. The model was able to extract the fissure features better than the spots and tooth marks. In the heat maps, the region of the fissures is completely red, but there are some blue or colorless parts in the regions with spots and tooth marks, which means that MobileNetV3Large cannot extract these two features either. Of course, the “lesser” performance here was compared to the fissures. For the mobile terminals, their accuracy was also acceptable. After all, the classification accuracy was basically around 90%. This phenomenon is reflected in Table 5.

The results presented above demonstrate that the models we used had good detection, segmentation, and classification performance.

## 5. Discussion

Tongue diagnosis is an important part of TCM inspection and is also the core component of TCM objectification. In recent years, there have been several studies on computer-aided tongue diagnosis systems, but most have been based on computers, which is inconvenient. Moreover, these systems usually focus on a certain part of the tongue, rather than conducting a complete intelligent tongue diagnosis process.

In this study, we combined deep learning and computer system technology to develop an intelligent tongue diagnosis system. Though the core of the system is tongue diagnosis, it has some other interesting parts: physical information collection and “asking doctors”, providing convenience to users to some extent. The framework of tongue diagnosis includes tongue detection (YOLOv5s6), tongue body segmentation (U-Net), and the classification (MobileNetV3Large) of tongue image features. The datasets used for training and testing included tongue images collected from 462 college students and a public dataset including 546 tooth-marked and 704 unmarked tongue images.

In our experiments, mAP@0.5:0.95 of YOLOv5s6 achieved a score of 97.15%, and in the model test, the tongue image detection box not only perfectly circled the tongue body but was also shown to have a high prediction probability of more than 93%, which proves that the network has satisfying effectiveness in detecting tongue images. The MIoU, U-Net’s evaluation metric, achieved a value of 97.86%, being almost 100%. Its effect can be demonstrated easily through the segmentation results and was almost exactly the same as that of the raw tongue images (there were only some subtle differences at the edges).

In the last step, MobileNetV3 was shown to have good accuracy for the tooth-marked tongue (93.33%), the spotted tongue (89.60%), and the fissured tongue (97.67%) images. The accuracy of the fissured tongue samples is higher than that of the other two types, and this phenomenon can be clearly seen from the heat maps created by Grad-CAM. This may be because there is an obvious difference between fissured tongues and non-fissured tongues in the tongue images, so this feature is easily extracted by the model. However, there is no obvious difference between the tooth-marked and spotted tongues or the unmarked and non-spotted tongues. In many cases, there is a small number of tooth-marked or spotted features in unmarked or non-spotted tongues. These two types of characteristics are more determined by the severity of the feature rather than the presence or absence of tooth marks or fissures in TCM.

Generally, the models achieved good performance in terms of tongue detection, tongue segmentation, and tongue feature classification.

## 6. Conclusions and Future Works

By combining the advanced deep learning algorithms and computer system technology in the field of image processing, we proposed an intelligent tongue diagnosis system based on the mobile terminal.

In this system, the tongue should be captured by the patient with a mobile phone, and the tongue image is initially detected and located through the object detection algorithm (YOLOv5s6). Then, the tongue image category is identified by the segmentation and classification algorithm (U-Net and MobileNetV3Large). Finally, a tongue diagnosis report is generated and fed back to the patient to achieve the effect of disease diagnosis. The experimental results and model evaluations prove that the performance, in terms of tongue detection, tongue segmentation, and tongue feature classification, gained satisfying results, and it has great value for intelligent and objective tongue diagnosis.

Although the system has made some progress, further work is still required. (1) The volunteers involved in this study were mainly college students, while “peel”, “curdy and greasy”, and “puffy and thin” tongue features usually appear among older adults and patients. Therefore, more comprehensive data should be collected from more diverse groups, such as patients, older adults, and people from different regions of China. (2) There is no authoritative quantitative standard for tongue color or fur color, and the system will quantify and classify these features to achieve a more comprehensive tongue diagnosis system in the future. (3) The sublingual veins stem from the base of the tongue and connect directly with the viscera, especially the heart and liver, which is also a factor that should be considered in tongue diagnosis, and this will be considered to combine tongue features in the future. (4) The diagnosis models can be further optimized by combining them with an excellent optimization algorithm, such as the firefly algorithm [56] and genetic algorithm [57].

## Figures and Tables

**Table 1:** Specifications for MobileNetV3Large.

Input	Operator	Exp Size	#out	SE	NL	s
$224^{2}\times 3$	conv2d	-	16	-	HS	2
$112^{2}\times 16$	bneck, 3×3	16	16	-	RE	1
$112^{2}\times 16$	bneck, 3×3	64	24	-	RE	2
$56^{2}\times 24$	bneck, 3×3	72	24	-	RE	1
$56^{2}\times 24$	bneck, 3×3	72	40	1	RE	2
$28^{2}\times 40$	bneck, 3×3	120	40	1	RE	1
$28^{2}\times 40$	bneck, 3×3	120	40	1	RE	1
$28^{2}\times 40$	bneck, 3×3	240	80	-	RE	2
$14^{2}\times 80$	bneck, 3×3	200	80	-	RE	1
$14^{2}\times 80$	bneck, 3×3	200	80	-	RE	1
$14^{2}\times 80$	bneck, 3×3	184	80	-	RE	1
$14^{2}\times 80$	bneck, 3×3	184	112	1	RE	1
$14^{2}\times 112$	bneck, 3×3	480	112	1	RE	1
$14^{2}\times 112$	bneck, 3×3	672	160	1	RE	2
$7^{2}\times 160$	bneck, 3×3	672	160	1	RE	1
$7^{2}\times 160$	bneck, 3×3	960	160	1	RE	1
$7^{2}\times 160$	conv2d, 1×1	960	960	-	HS	1
$7^{2}\times 960$	avg pool, 7×7	-	-	-	-	1
$1^{2}\times 960$	conv2d, 1×1	-	1280	-	HS	1
$1^{2}\times 1280$	conv2d, 1×1	-	k	-	-	1

**Table 2:** The tongue fur and body features.

Tongue	Feature				
Fur	Thin and thick	Moist and dry	Curdy and greasy	Peeled	True and false
Body	Puffy and thin	Old and tender	Tooth-marked	Fissured	Spotted

**Table 3:** The number of training and testing data for each task.

Task	Number	Train	Test
Tongue image detection	462	370	92
Tongue body segment	462	370	92
Fissured/not fissured	170/292	236/234	34/58
Tooth-marked/not marked	546/704	437/564	109/140
Spotted/not spotted	210/252	168/202	42/34

**Table 4:** The precise configuration of the experiments.

Task	Model	Epoch	Batch Size
Tongue detection	YOLOv5s6	40	16
Tongue region segment	U-Net	100	16
Fissured or not fissured	MobileNetV3	200	64
Tooth-marked or not marked	MobileNetV3	200	64
Spotted or not spotted	MobileNetV3	200	64

**Table 5:** The best results of different datasets.

Feature	Tooth-Marked	Spotted	Fissured
Accuracy	93.33%	89.60%	97.67%
F1-score	92.61%	82.93%	96.55%
Recall	92.16%	78.46%	96.55%
Precision	93.07%	87.93%	96.55%
Specificity	94.31%	94.89%	98.25%

## Data Availability

The data of tooth marks are available from https://www.kaggle.com/datasets/clearhanhui/biyesheji (accessed on 10 September 2019). The other data presented in this study are available on request from the corresponding author. These data are not publicly available due to this data being supplied by the China Academy of Chinese Medical Sciences.

## References

[1] Cyranoski D. (2018). Why Chinese medicine is heading for clinics around the world. Nature.

[2] Ozgursoy O.B., Ozgursoy S.K., Tulunay O., Kemal O., Akyol A., Dursun G. (2009). Melkersson-Rosenthal syndrome revisited as a misdiagnosed disease. Am. J. Otolaryngol..

[3] Avraham K.B., Schickler M., Sapoznikov D., Yarom R., Groner Y. (1988). Down’s syndrome: Abnormal neuromuscular junction in tongue of transgenic mice with elevated levels of human Cu/Zn-superoxide dismutase. Cell.

[4] Farman A.G. (1976). Atrophic lesions of the tongue: A prevalence study among 175 diabetic patients. J. Oral Pathol. Med..

[5] Wang X., Zhang D. (2013). A high quality color imaging system for computerized tongue image analysis. Expert Syst. Appl..

[6] LeCun Y., Bengio Y., Hinton G. (2015). Deep learning. Nature.

[7] Guo Y., Liu Y., Oerlemans A., Lao S., Wu S., Lew M.S. (2016). Deep learning for visual understanding: A review. Neurocomputing.

[8] Razzak M.I., Naz S., Zaib A. (2018). Deep learning for medical image processing: Overview, challenges and the future. Classification in BioApps.

[9] Zhou J., Zhang Q., Zhang B. (2021). An automatic multi-view disease detection system via collective deep region-based feature representation. Future Gener. Comput. Syst..

[10] Jiang T., Guo X.J., Tu L.P., Lu Z., Cui J., Ma X.X., Hu X.J., Yao X.H., Cui L.T., Li Y.Z. (2021). Application of computer tongue image analysis technology in the diagnosis of NAFLD. Comput. Biol. Med..

[11] Gholami E., Tabbakh S.R.K., Kheirabadi M. (2020). Proposing method to Increase the detection accuracy of stomach cancer based on colour and lint features of tongue using CNN and SVM. arXiv.

[12] Tang Q., Yang T., Yoshimura Y., Namiki T., Nakaguchi T. (2020). Learning-based tongue detection for automatic tongue color diagnosis system. Artif. Life Robot..

[13] Zhou C., Fan H., Li Z. (2019). Tonguenet: Accurate localization and segmentation for tongue images using deep neural networks. IEEE Access.

[14] Zhou J., Zhang Q., Zhang B., Chen X. (2019). TongueNet: A precise and fast tongue segmentation system using U-Net with a morphological processing layer. Appl. Sci..

[15] Hou J., Su H.Y., Yan B., Zheng H., Sun Z.L., Cai X.C. (2017). Classification of tongue color based on CNN. Proceedings of the 2017 IEEE 2nd International Conference on Big Data Analysis (ICBDA).

[16] Li X., Zhang Y., Cui Q., Yi X., Zhang Y. (2019). Tooth-Marked Tongue Recognition Using Multiple Instance Learning and CNN Features. IEEE Trans. Cybern..

[17] Vukotic A., Goodwill J. (2011). Introduction to Apache Tomcat 7. Apache Tomcat 7.

[18] Ronneberger O., Fischer P., Brox T. (2015). U-net: Convolutional networks for biomedical image segmentation. Proceedings of the International Conference on Medical Image Computing and Computer-Assisted Intervention.

[19] Howard A., Sandler M., Chu G., Chen L.C., Chen B., Tan M., Wang W., Zhu Y., Pang R., Vasudevan V. Searching for mobilenetv3. Proceedings of the IEEE/CVF International Conference on Computer Vision.

[20] Redmon J., Divvala S., Girshick R., Farhadi A. You Only Look Once: Unified, Real-Time Object Detection. Proceedings of the 2016 IEEE Conference on Computer Vision and Pattern Recognition (CVPR).

[21] Redmon J., Farhadi A. YOLO9000: Better, Faster, Stronger. Proceedings of the 2017 IEEE Conference on Computer Vision and Pattern Recognition (CVPR).

[22] Redmon J., Farhadi A. (2018). Yolov3: An incremental improvement. arXiv.

[23] Bochkovskiy A., Wang C.Y., Liao H.Y.M. (2020). Yolov4: Optimal speed and accuracy of object detection. arXiv.

[24] Liu W., Anguelov D., Erhan D., Szegedy C., Reed S., Fu C.Y., Berg A.C. (2016). Ssd: Single shot multibox detector. Proceedings of the European Conference on Computer Vision.

[25] Fu C.Y., Liu W., Ranga A., Tyagi A., Berg A.C. (2017). Dssd: Deconvolutional single shot detector. arXiv.

[26] Liu S., Huang D., Wang Y. Receptive field block net for accurate and fast object detection. Proceedings of the European Conference on Computer Vision (ECCV).

[27] Zhang S., Wen L., Bian X., Lei Z., Li S.Z. Single-shot refinement neural network for object detection. Proceedings of the IEEE Conference on Computer Vision and Pattern Recognition.

[28] Girshick R., Donahue J., Darrell T., Malik J. Rich feature hierarchies for accurate object detection and semantic segmentation. Proceedings of the IEEE Conference on Computer Vision and Pattern Recognition.

[29] Girshick R. Fast R-CNN. Proceedings of the 2015 IEEE International Conference on Computer Vision (ICCV).

[30] Ren S., He K., Girshick R., Sun J. (2017). Faster R-CNN: Towards Real-Time Object Detection with Region Proposal Networks. IEEE Trans. Pattern Anal. Mach. Intell..

[31] He K., Gkioxari G., Dollár P., Girshick R. Mask R-CNN. Proceedings of the 2017 IEEE International Conference on Computer Vision (ICCV).

[32] He K., Zhang X., Ren S., Sun J. (2015). Spatial Pyramid Pooling in Deep Convolutional Networks for Visual Recognition. IEEE Trans. Pattern Anal. Mach. Intell..

[33] Lin T.Y., Dollár P., Girshick R., He K., Hariharan B., Belongie S. Feature Pyramid Networks for Object Detection. Proceedings of the 2017 IEEE Conference on Computer Vision and Pattern Recognition (CVPR).

[34] Liu S., Qi L., Qin H., Shi J., Jia J. Path Aggregation Network for Instance Segmentation. Proceedings of the 2018 IEEE/CVF Conference on Computer Vision and Pattern Recognition.

[35] Long J., Shelhamer E., Darrell T. Fully convolutional networks for semantic segmentation. Proceedings of the 2015 IEEE Conference on Computer Vision and Pattern Recognition (CVPR).

[36] Chen L.C., Barron J.T., Papandreou G., Murphy K., Yuille A.L. Semantic Image Segmentation with Task-Specific Edge Detection Using CNNs and a Discriminatively Trained Domain Transform. Proceedings of the 2016 IEEE Conference on Computer Vision and Pattern Recognition (CVPR).

[37] Chen L.C., Papandreou G., Kokkinos I., Murphy K., Yuille A.L. (2018). DeepLab: Semantic Image Segmentation with Deep Convolutional Nets, Atrous Convolution, and Fully Connected CRFs. IEEE Trans. Pattern Anal. Mach. Intell..

[38] Chen L.C., Papandreou G., Schroff F., Adam H. (2017). Rethinking atrous convolution for semantic image segmentation. arXiv.

[39] Chen L.C., Zhu Y., Papandreou G., Schroff F., Adam H. Encoder-decoder with atrous separable convolution for semantic image segmentation. Proceedings of the European Conference on Computer Vision (ECCV).

[40] Wang Y.P., Jheng Y.C., Sung K.Y., Lin H.E., Hsin I.F., Chen P.H., Chu Y.C., Lu D., Wang Y.J., Hou M.C. (2022). Use of U-Net Convolutional Neural Networks for Automated Segmentation of Fecal Material for Objective Evaluation of Bowel Preparation Quality in Colonoscopy. Diagnostics.

[41] Simonyan K., Zisserman A. (2014). Very deep convolutional networks for large-scale image recognition. arXiv.

[42] He K., Zhang X., Ren S., Sun J. Deep Residual Learning for Image Recognition. Proceedings of the 2016 IEEE Conference on Computer Vision and Pattern Recognition (CVPR).

[43] He K., Zhang X., Ren S., Sun J. (2016). Identity mappings in deep residual networks. Proceedings of the European Conference on Computer Vision.

[44] Zagoruyko S., Komodakis N. (2016). Wide residual networks. arXiv.

[45] Xie S., Girshick R., Dollár P., Tu Z., He K. Aggregated Residual Transformations for Deep Neural Networks. Proceedings of the 2017 IEEE Conference on Computer Vision and Pattern Recognition (CVPR).

[46] Howard A.G., Zhu M., Chen B., Kalenichenko D., Wang W., Weyand T., Andreetto M., Adam H. (2017). Mobilenets: Efficient convolutional neural networks for mobile vision applications. arXiv.

[47] Sandler M., Howard A., Zhu M., Zhmoginov A., Chen L.C. MobileNetV2: Inverted Residuals and Linear Bottlenecks. Proceedings of the 2018 IEEE/CVF Conference on Computer Vision and Pattern Recognition.

[48] Powers D.M. (2020). Evaluation: From precision, recall and F-measure to ROC, informedness, markedness and correlation. arXiv.

[49] Russell B.C., Torralba A., Murphy K.P., Freeman W.T. (2008). LabelMe: A database and web-based tool for image annotation. Int. J. Comput. Vis..

[50] Tzutalin D. (2015). LabelImg. GitHub Repos..

[51] Li Q., Guo H., Luo L., Wang X. (2022). Automatic Mapping of Karez in Turpan Basin Based on Google Earth Images and the YOLOv5 Model. Remote Sens..

[52] Dong X., Yan S., Duan C. (2022). A lightweight vehicles detection network model based on YOLOv5. Eng. Appl. Artif. Intell..

[53] Murphy K.P. (2012). Machine Learning: A Probabilistic Perspective.

[54] Zheng Z., Wang P., Liu W., Li J., Ye R., Ren D. Distance-IoU loss: Faster and better learning for bounding box regression. Proceedings of the AAAI Conference on Artificial Intelligence.

[55] Selvaraju R.R., Cogswell M., Das A., Vedantam R., Parikh D., Batra D. Grad-CAM: Visual Explanations from Deep Networks via Gradient-Based Localization. Proceedings of the 2017 IEEE International Conference on Computer Vision (ICCV).

[56] Bacanin N., Stoean R., Zivkovic M., Petrovic A., Rashid T.A., Bezdan T. (2021). Performance of a Novel Chaotic Firefly Algorithm with Enhanced Exploration for Tackling Global Optimization Problems: Application for Dropout Regularization. Mathematics.

[57] Malakar S., Ghosh M., Bhowmik S., Sarkar R., Nasipuri M. (2020). A GA based hierarchical feature selection approach for handwritten word recognition. Neural Comput. Appl..

